# Adapting and Implementing a School-Based “Implementation Intentions” Program Within FRESHAIR4Life to Prevent Smoking Initiation Among Adolescents in Greece: A Study Protocol

**DOI:** 10.3390/healthcare14070938

**Published:** 2026-04-03

**Authors:** Izolde Bouloukaki, Antonios Christodoulakis, Sevasti Peraki, Floor A. Van Den Brand, Faraz Siddiqui, Theodoros Krasanakis, Antonia Aravantinou-Karlatou, Purva Abhyankar, Siân Williams, Julia van Koeveringe, Rianne MJJ van der Kleij, Ioanna Tsiligianni

**Affiliations:** 1 Department of Social Medicine, School of Medicine, University of Crete, 71500 Heraklion, Greece; christodoulakisa@uoc.gr (A.C.); sevastiperaki@gmail.com (S.P.); medp2012148@med.uoc.gr (T.K.); toniakoinerg2@gmail.com (A.A.-K.); i.tsiligianni@uoc.gr (I.T.); 2 Department of Family Medicine, Maastricht University (CAPHRI), 6200 Maastricht, The Netherlands; f.vandenbrand@maastrichtuniversity.nl (F.A.V.D.B.); julia.vankoeveringe@maastrichtuniversity.nl (J.v.K.); 3 Department of Health Sciences, University of York, York YO10 5DD, UK; faraz.siddiqui@york.ac.uk; 4 Usher Institute, College of Medicine and Veterinary Medicine, University of Edinburgh, Edinburgh EH16 4UX, UK; purva.abhyankar@ed.ac.uk; 5 International Primary Care Respiratory Group, Edinburgh EH2 4JN, UK; sian@ipcrg.org; 6 Department of Public Health and Primary Care, Leiden University Medical Center, 2333 Leiden, The Netherlands; m.j.j.van_der_kleij-van_der_sluis@lumc.nl; 7 Department of Primary Care and Population Health, Medical School, University of Nicosia, 2417 Nicosia, Cyprus

**Keywords:** Implementation Intentions, FRESHAIR4Life, smoking prevention, tobacco use, susceptibility, adolescents, If–Then

## Abstract

**Background**: Most individuals develop smoking habits in adolescence, highlighting the need for a smoking prevention program targeted at this age group. The use of “Implementation Intentions” (If-Then plans) about how to refuse a cigarette combined with anti-smoking messages has been shown to be effective in the UK. However, there is a scarcity of data regarding school-based smoking prevention interventions among adolescents available to countries with high tobacco consumption rates, like Greece. **Objectives**: To describe the cultural adaptation procedure and the evaluation protocol for the school-based “Implementation Intentions” program aimed at reducing tobacco use susceptibility among Greek adolescents aged 13–16 in school settings. **Methods**: The present study is part of the EU-funded FRESHAIR4Life Program. We will use a mixed-methods approach with a pre- and post-intervention design in six conveniently selected secondary schools in Heraklion, Crete, Greece, to measure the intervention’s Reach, Effectiveness, Adoption, Implementation, and Maintenance using the RE-AIM framework. The study plans to involve three Master Trainers (MTs), 20–25 school teachers (to be trained by the MTs), and approximately 480 students. Participating schools will receive the “Implementation Intentions” intervention, which is based on a goal-setting technique where individuals commit to perform a particular behavior when a specific context arises. The study will consist of five sequential phases: Phase I involves training three Master Trainers (MTs) using the International Primary Care Respiratory Group (IPCRG’s) Teach-the-Teacher (TtT) curriculum, specifically focused on the implementation of our intervention. In Phase II, workshops will be held to co-create and culturally adapt the intervention. Phase III will involve teachers trained by MTs on delivering the intervention. In Phase IV, teachers will deliver the intervention among students in their schools. Data will be collected pre- and post-intervention through surveys, session logs, fidelity observations, feedback forms, and follow-up interviews or focus groups (Phase V). Quantitative data will be analyzed descriptively and by using paired t-tests and multiple linear regression analyses, while qualitative data will undergo thematic analysis. **Discussion**: The study protocol’s potential benefits extend beyond educating Greek adolescents on the risks associated with smoking. Active participation will empower and motivate young people to make informed, healthy choices. We expect the results could help create more effective, context-specific interventions, support policy changes aimed at decreasing the prevalence of adolescent smoking in Crete, Greece, and potentially be used by other countries as well.

## 1. Introduction

Tobacco use (TU) constitutes an important modifiable risk factor for Non-Communicable Diseases (NCDs), representing 70% of all mortality globally [1]. While NCDs usually emerge in adulthood, early smoking initiation during adolescence increases the probability of the development of NCDs at a younger age [2,3]. This also increases the chances of retardation of lung growth and optimal lung function, experiencing adverse respiratory symptoms such as wheezing and nighttime coughing and the risk of developing asthma during adolescence [4,5,6,7]. These short-term respiratory problems pose a risk to current quality of life and may contribute to an increased likelihood of school absenteeism from temporary illness or breathing difficulties [8]. Furthermore, the developing adolescent brain is more vulnerable to nicotine and addiction, which substantially increases the risk of greater dependence and heavier smoking later on [9]. TU during adolescence is also associated with increased risk of adverse mental health outcomes and psychiatric disorders, as well as higher prevalence of marijuana use, illicit drug use and alcohol use [10,11,12].

Adolescence is the most critical stage for tobacco consumption, as young individuals are more vulnerable to social influences, with 90% of smokers initiating before the age of 19 [13,14]. Globally, approximately 12% of adolescents aged 13–15 years old report the use of one or more types of tobacco products, and in Europe this rate reaches 12.6% [15]. A significant concern is the ongoing worldwide rise in e-cigarette and nicotine pouch use among young people, posing new public health issues and detrimental effects on adolescent well-being [16,17].

Smoking rates in Greece are higher than in other European Union countries, with a prevalence of 15% among 15–16 year olds [18]. Without effective interventions, the high rates of smoking among adolescents will escalate the public health burden, and an estimated 5.6 million of today’s youth under the age of 18 are projected to experience premature mortality from TU diseases [19].

Several primary care behavioral interventions, including education or brief counseling, have been implemented to reduce TU in low-, middle- and high-income countries that target adolescents with a primary focus on smoking cessation [20,21], yet these measures have shown limited long-term success [21,22,23,24]. However, beyond the importance of designing and implementing interventions to help smokers quit, it is crucial to work towards preventing the initiation of young people into this addiction. The focus of prevention programs should be on educating adolescents who have never smoked to increase their knowledge, develop positive attitudes towards not smoking, build self-confidence, and give them the skills to decline the use of tobacco products. Additionally, programs should prevent adolescents already experimenting with TU from continuing [25].

In this context, schools present an excellent opportunity to introduce healthy lifestyle programs, such as those aimed at preventing TU, because they have access to a large and diverse student population at a key developmental stage, at a location where these students spend much of their day. Furthermore, these settings have proven useful for facilitating changes in numerous health behaviors [26]. School-based programs, such as the INTENT program from the University of Leeds in the UK [27], use “Implementation Intentions” (specific “if…then” plans) and have shown promise in Randomized Clinical Trials by reducing smoking initiation by 6.5% among 15–16-year-olds, four years on from the start of the intervention, and 25.6% of the students in the program were less likely to report having ever smoked compared to those who did not take part [27,28,29]. By employing “Implementation Intentions”, the program supports young people in resisting tobacco products offers by increasing their motivation to abstain from smoking and then helping them develop specific personalized plans/strategies to act on that motivation, in situations where they are offered tobacco products [30,31].

Despite promising indications of school-based interventions, few rigorous evaluations exist, and systematic reviews and meta-analyses have indicated that the impact of school-based smoking programs on adolescent smoking behavior has been less significant than initially expected [32,33,34,35,36]. Nevertheless, it is important to acknowledge that current programs often demonstrate inadequate local cultural adaptation and have mainly been evaluated in high-income settings, which raises questions about their general applicability to other regions. For this reason, implementation research on the tailored, multidisciplinary NCD prevention package ‘FRESHAIR4Life: Targeting tobacco and air pollution exposure in mid to late adolescents in disadvantaged populations’, an EU-funded Horizon Europe project, was initiated to adapt and implement targeted interventions for prevention and cessation in countries with varied economies that experience high TU rates, such as Greece, Kyrgyz Republic, Pakistan, and Romania [37]. Specifically in Greece, there is a scarcity of data regarding school-based smoking prevention interventions among adolescents [38,39]. In previous research, student volunteers were tasked with developing anti-smoking audiovisual materials, which they presented and reviewed with all participating students. This approach, while significantly limiting experimental smoking among adolescents, did not affect their future smoking intentions, attitudes towards smoking, or knowledge about it [38,39]. Utilizing ancient Greek literature, myths, and poems for educational purposes in a more recent study showed potential in improving smoking-related knowledge, strengthening anti-smoking attitudes, and decreasing the likelihood of initiating or continuing smoking [38,39]. The “I do not smoke, I exercise” school program showed promising results in terms of participant knowledge, perceived control, and overall satisfaction; however, its impact on participants’ smoking behavior, students’ attitudes toward smoking, and their intention to smoke was limited [40].

The aforementioned findings have important implications for smoking prevention programs in Greece and highlight the need for further exploration of tobacco control interventions in Greek schools. Therefore, the aim of this study protocol is to describe the cultural adaptation procedure and the proposed implementation and evaluation of the school-based “Implementation Intentions” program aimed at reducing TU susceptibility among Greek adolescents aged 13–16 in school settings. The specific research questions of this study are:

- To what extent is the Greek adaptation of the INTENT program associated with changes in tobacco use susceptibility and related attitudes among adolescents aged 13–16?
- How do key stakeholders, including teachers and a representative group of adolescents, perceive the feasibility, acceptability, and potential effectiveness of the INTENT program?”

## 2. Materials and Methods

The present protocol (version 7, dated 28 February 2025) has been based on the Standard Protocol Items Recommendations for Interventional Trials (SPIRIT) (Supplementary Materials Table S1) [41].

### 2.1. Study Design

**Figure 1:**
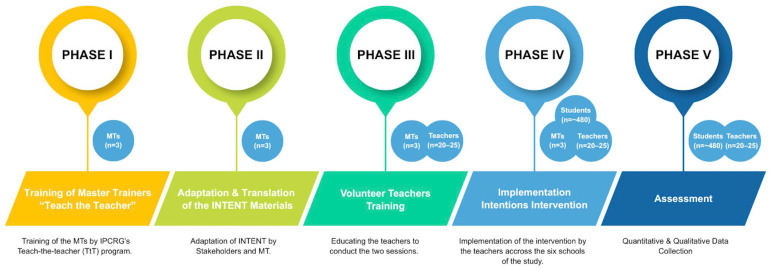
Outline of the phases of the study.

The present protocol is part of the EU-funded FRESHAIR4Life Program. This protocol outlines the five phases (Figure 1) of the adaptation, preparation, and implementation plan of a smoking prevention intervention based on “Implementation Intentions” from the INTENT project from the UK [27]. The initial phase (Phase I) will involve training three Master Trainers (MTs) using the International Primary Care Respiratory Group (IPCRG’s) Teach-the-Teacher (TtT) curriculum, specifically focused on the implementation of our intervention [42,43]. In the second phase (Phase 2), these trainers will then adapt the UK’s INTENT curriculum, employing the Actor, Action, Context, Target, Time (AACTT) framework to plan the key activities for training the school teachers [44]. Subsequently, in the third phase (Phase III), they will train school teachers from six schools in Heraklion (three middle and three high schools) to deliver (Phase IV) and assess (Phase V) the effect of the intervention on adolescents aged 13–16. It is important to acknowledge that these schools were selected in collaboration with local educational authorities, taking into consideration diversity in student populations based on socioeconomic context and school size and aligning with FRESHAIR4Life’s aims of accessibility and cultural relevance (Context-Specific Tailoring). Specifically, all six public secondary schools are situated in urban and peri-urban areas of Heraklion, Crete. The selection process, conducted in conjunction with the Directorate of Secondary Education of the Heraklion Prefecture, considered factors such as differences in school size (student enrollment) and the socioeconomic diversity of students, as indicated by neighborhood characteristics (since students enroll at the school nearest to their home). This approach also aligns with FRESHAIR4Life’s commitment to addressing the needs of disadvantaged populations. Teacher training will be offered online and in-person at the schools, and this school-centered intervention will be conducted during school hours. Outcomes will be evaluated using a mixed-methods approach (quantitative and qualitative data), guided by the RE-AIM (Reach, Effectiveness, Adoption, Implementation, Maintenance) framework, with data collection points at baseline, mid-point (between sessions), and post-implementation [45].

### 2.2. Study Population

The participants in this study will be categorized into three groups: the MTs, the teachers and the adolescents. Firstly, a selected group of three experienced educators/trainers who will act as MTs will be recruited. These individuals will be responsible for training the school teachers and overseeing the fidelity of the intervention delivery. MTs will undergo training sessions on tobacco prevention, facilitation techniques, and the “If–Then” planning methodology. Based on their new knowledge and skills, MTs will conduct training sessions and provide feedback and support to teachers throughout the implementation phase. Secondly, school teachers will be selected to participate in the intervention. These teachers will be trained by the MTs to deliver the tobacco prevention intervention to the adolescent participants. All teachers from the participating schools (approximately n = 250) will be invited to participate in the program. However, as this is a pilot intervention, the first four to five teachers from each school will be accepted to participate in the study, resulting in a total of 20 to 25 participants. The first-come, first-served approach was primarily adopted for pragmatic reasons, aligning with the goals of a pilot implementation study. Firstly, as this is an initial feasibility and implementation protocol, the main objective is to determine if the intervention can be successfully delivered by willing teachers in a real-world setting, rather than to recruit a perfectly representative sample. Secondly, restricting participation to 4–5 teachers per school creates a practical and manageable group size for the intensive training provided. Nevertheless, and considering that each classroom includes around 20 students, we anticipate that the intervention will reach approximately 480 adolescents.

#### 2.2.1. Selection and Preparation of Country MTs (Phase I)

Three MTs will be selected with a strong background in public health, applying facilitation techniques and addressing the diverse challenges faced by adolescents. The selected MTs will be required to be motivated and tobacco-free individuals with a proven dedication to adolescent engagement. MTs need to have completed the IPCRG’s Teach-the-Teacher (TtT) program adapted to facilitate the “Implementation Intentions” intervention. The program was conducted by IPCRG from November to December 2024 and featured interactive online sessions covering numerous topics, such as “Behaviour Change with a Focus on Adolescents”, “Teaching Skills (Learning Needs, Objectives, and Methods)”, and “The If-Then Approach and Peer Learning”. [46]. This training empowered participants to more effectively train the school teachers by transferring both the knowledge and the pedagogical techniques necessary for delivering our intervention.

Given the overlapping roles of Master Trainers in adaptation, training, and evaluation, they will document their assumptions, and roles will be clarified so that those adapting the training are not solely responsible for its evaluation. Specifically, all MTs will keep a structured adaptation logbook detailing each procedure (adaptation, training of teachers, evaluation, etc.). Moreover, MTs will focus primarily on delivery and teacher training and support, while different research team members will conduct data collection, evaluation, and analysis. Furthermore, an independent FRESHAIR4Life member will oversee all processes, receive regular updates from country team members, assess their progress, and offer feedback.

#### 2.2.2. Selection of School Teachers

Teachers will be invited to participate in the intervention through outreach visits by the MTs at the schools. These visits will include face-to-face meetings with headmasters and teachers and lectures/presentations in six schools. To be eligible, teachers must be employed at a participating school, willing to attend both training sessions, willing to deliver the program to their classes, and able to communicate effectively in Greek. No exclusion criteria were predetermined for teachers.

#### 2.2.3. Selection of Students

The intervention’s target demographic is middle- and high- school students (age: 13–16 years), with the participating school teachers acting as implementers. Initially, lectures will be scheduled at all selected schools to present information on smoking prevalence, effects, and the planned intervention with headmasters, teachers, and parents invited. These lectures, presented by the MTs, will inform and clarify the intervention to participating teachers and parents, thereby encouraging parental support for student participation. Then, the headmaster or a representative teacher from each school will inform all parents/legal guardians of the adolescents about the program. The students to be included should be enrolled in the classes of the trained teachers (implementors). Each student will be given an information sheet (outlining the study), an informed consent form for their legal guardian to sign, and an assent form for them to sign. All students in the participating classes will receive the intervention, though they have the option to decline participation in its evaluation. To be eligible for the evaluation of the intervention, participants must have the ability to understand the Greek language, agree to participate in the evaluation (by signing the informed assent) and to have the consent of a parent or a guardian (by signing the informed consent). Students who do not provide signed assent and consent forms will be excluded from the evaluation and will engage in a separate activity with the headmaster (smoking prevention-related) outside the classroom during the evaluation.

### 2.3. Adaptation of the Material by the MTs (Phase II)

The “If–Then” intervention was originally developed for adolescents in the UK, aged 11–15, with sessions lasting two hours per year for four consecutive school years, delivered in school settings by teachers [27,29,31,47]. However, for its implementation in Greece and for our study, several modifications will be required to accommodate the distinct sociocultural context. In addition, the target age range was adapted from the original 11–15 (Intent’s age range) to 13–16 years. This was intentional, since in the structure of the Greek secondary school system, middle school enrolls students aged 13–15, while high school covers ages 15–18. As students aged 11–12 do not attend Greek secondary schools, they are not accessible within a middle school-based delivery model, making the 13–16 range both appropriate and operationally necessary. The overall cultural adaptation process was guided by the principles of systematic intervention adaptation (including stakeholder engagement, iterative review, and piloting), while AACTT (Actor, Action, Context, Target, Time) was applied specifically to specify and articulate the key behaviors targeted by the intervention [44]. The adaptation process will involve a collaborative effort with key stakeholders, including teachers, behavioral experts, and a representative group of adolescents. For this reason, a meeting with the three selected MTs, one secretary (for log keeping), and at least four (4) middle school teachers, four (4) high school teachers, and ten (10) adolescents (14–16 years old), will be held to adapt the materials. The meeting agenda will include the timing of the implementation, the number and duration of sessions, the review and selection of the most appropriate INTENT materials and pilot testing of the material. Teachers and students will jointly discuss and agree on both the selection of the materials and the rationale behind these choices. After a comprehensive review of all materials, the participants will discuss the adaptation process. An adaptation log will be used to document decisions about changes, including the proposed modifications, the justification for them, and the stakeholders who will have participated. These proposals will then be discussed in stakeholder meetings, with agreements or concerns captured in brief minutes. Decisions will be finalized via consensus and documented in a protocol to guarantee transparency and traceability.

The research team (co-authors of this paper), to facilitate the adaptation process, has already discussed and agreed that the original UK session structure should be reduced from eight to two sessions (80–90 min each), and incorporate language and visual adjustments while expanding session content to encompass TU vaping and nicotine pouch use in addition to cigarette smoking, due to the high prevalence of use by Greek adolescents. Furthermore, a written supplementary resource will be developed to guide teachers in addressing and supporting students with existing TU who may wish to quit.

### 2.4. The Implementation

The intervention will be conducted in two phases (Table 1). The first phase will prepare the participating teachers to utilize the material. The second phase will involve the implementation of the material by the teachers in their classrooms.

#### 2.4.1. Preparation Phase—Teaching the Volunteer Teachers (Phase III)

Teachers will participate in an intensive training program led by the selected MTs. The training will be divided into two separate structured training sessions, one held online and one face-to-face (Table 2). Both of these sessions will be delivered by the MTs, approximately one to two weeks prior to implementation. The first session (about one hour), will be held online by the MTs, who will present teachers with all the adapted and translated INTENT materials, including session guides, presentations (power point format), explanatory videos, and relevant resources, through the “Google Classroom” platform. Specifically, the MTs will introduce the program’s targets, the importance of addressing adolescent TU, and the theoretical background behind the “If–Then” implementation (e.g., “If I’m offered a cigarette, then I’ll say I don’t smoke because it affects my asthma”), while also answering questions [46]. The second session will consist of a one-hour workshop held in the school environment in each of the six participating schools (4–5 teachers in each school), where MTs will guide teachers through all the necessary techniques for a successful implementation of the intervention. The workshop will build on the foundation established in the first session, providing a hands-on experience to refine teachers’ abilities to deliver the intervention with active learning techniques. These techniques usually include open-ended questioning and active listening, practicing in pairs. Furthermore, teachers will participate in role-plays addressing common challenges of adolescents, such as peer pressure, vaping myths and truths, or students who face difficulty engaging with others. After each role-play, the group participates in a debrief, discussing what worked well and identifying areas for improvement. Each teacher will deliver a short practice segment of the intervention and will receive structured feedback from both MTs and colleagues, and will repeat the activity to refine pacing, clarity, and interaction skills, incorporating the feedback they received.

#### 2.4.2. Implementation Phase of the Study—Teachers Utilizing the Intervention at Their Classrooms (Phase IV)

The second phase of the intervention will be based on the deliverance of the “Implementation Intentions” intervention by the teachers at the schools. The implementation, as mentioned above, will be conducted in six different schools and will consist of two structured sessions (Table 3). Both sessions, each lasting two school hours (~90 min), will be delivered by trained teachers, scheduled approximately two months apart. MTs will oversee all sessions and assist teachers where needed. In the first session, teachers will introduce our intervention to students. Introducing our intervention, teachers will explain that through this program students will have the opportunity to explore the factors in their life that may influence them to smoke. Teachers will highlight the fact that committing to do something by putting this commitment down on paper is much more likely to result in success, by creating a “Personal Plan (PP)”. This session includes interactive conversations with students addressing myths and truths about TU and the long-term effects of initiating smoking, as well as quizzes regarding TU. The first session will conclude with a written exercise outlining each student’s refusal tactics, followed by the development of their Personal Plans (PPs). The second session aims to strengthen students’ personal resilience and highlight the complexity of external influences on their decision to remain smoke-free. Moreover, teachers will examine what students can recall from the previous lecture. Students will be informed about how smoking interplays with stress and once again, they will create their PP. It should be noted that both quantitative and qualitative data will be collected by the MTs, before, during and after the intervention.

### 2.5. Data Collection (Phase V)

Data collection for this study will be organized around the intervention. First, students and teachers will complete quantitative questionnaires. These will be given at two points, (1) before the intervention (baseline) and (2) after the two sessions (post-intervention follow-up). Second, fidelity and implementation-related data will be collected during the intervention (session logs and observational checklists). These will be collected between the two sessions. Third, qualitative data will be collected after the intervention. This will involve semi-structured interviews and focus groups with participants (students and teachers). Moreover, team-members (other than MTs) will collect the data at each phase of the intervention. As stated, both quantitative (questionnaires) and qualitative data (interviews and FGs) will be collected for this study. The questionnaires will be developed for this and will consist of closed and open questions presented in a structured format. Specifically, close-ended questions will be either Likert-type (rated 1-almost never to 5-almost always) or dichotomous (yes or no). Each question will yield a score, and these scores will be summed for a total. Higher total scores will indicate a greater level of the measured variable. The estimated time for completing both the questionnaires and the interview is approximately 60 min.

#### 2.5.1. Quantitative Data Collection Students

Quantitative data from students will be collected using two structured, self-administered questionnaires developed for this study at two timepoints, baseline (before the delivery of the sessions) and follow-up (after the delivery of the two sessions) (Table 4). The baseline questionnaire will assess socio-demographic characteristics, family environment, cigarette use, e-cigarette use, nicotine pouch use, exposure to secondhand smoke, exposure to air pollution, respiratory symptoms, and the impact these factors have on students’ productivity. Tobacco-related sections will include detailed measures of ever-use of tobacco products, past-30-day use of tobacco products, tobacco offers received from peers, susceptibility and curiosity towards tobacco use, intentions (to initiate smoking, quit smoking or try smoking), and perceived harm from three product categories: cigarettes, e-cigarettes, and nicotine pouches. The follow-up questionnaire will include 50 items. This questionnaire will reassess tobacco use and susceptibility measures to allow for pre- and post-intervention comparisons. There will be a section which will assess refusal capacity, the perceived usefulness of the two sessions, and students’ reactions to tobacco offers. For both questionnaires, students will rate their agreement on a four-point Likert scale, ranging from 1 (probably not) to 4 (probably yes).

#### 2.5.2. Quantitative Data Collection Teachers

Quantitative implementation data from teachers will be collected using two structured questionnaires at two timepoints, baseline (pre-intervention/before the delivery of the sessions) and follow-up (post-intervention/after the delivery of the two sessions) (Table 5). The first questionnaire will be the teacher self-assessment questionnaire, and it will be completed by the trained teachers. This questionnaire will include 20 items evaluating preparation, familiarity with the intervention, alignment with school values, feasibility, confidence, and resource needs. Moreover, it will use open questions regarding student engagement, and the quality of Personal Plan implementation. For both questionnaires, teachers will rate their agreement on a four-point Likert scale, ranging from 1 (disagree) to 4 (agree).

#### 2.5.3. Qualitative Data Collection for Teachers, MTs, and Students

Qualitative data from students (Table 4), MTs, and teachers (Table 5) will be collected through semi-structured interviews, focus groups and open-ended written questions, post-intervention. Following the intervention, MTs will organize semi-structured interviews or focus groups, inviting participation from students and teachers (at least 3–4 students and 2–3 teachers from each school), separately. These interviews/focus groups will explore the perceived effectiveness, cultural relevance of intervention, ease of understanding, and areas for improvement. Regarding the MTs, a FRESHAIR4Life team member will interview them in a semi-structured format about their experience organizing the intervention, any difficulties they encountered, and suggestions for improvement in future implementations.

### 2.6. Sample Size

A sample size calculation was conducted using G*Power software (version 3.1) for the most complex analysis planned in this study, multiple linear regression examining the relationship between tobacco-related behaviors (initiation, use, and susceptibility) and key predictors (adolescent attitudes and perceptions about smoking) [48,49]. For a medium effect size (f^2^ = 0.15), an alpha level of 0.05, and a desired power of 0.80, the analysis indicated that a minimum of 77 participants would be required. Taking into account a potential 20% attrition rate and considering that the most complex analysis requires a sample size of 77 participants, the minimum target sample size was set at 93 participants; however, we expect that at least 450 adolescents will participate in our study.

### 2.7. Data Analysis

Quantitative data will be analyzed using the IBM SPSS 29 program. Descriptive statistics will summarize participant characteristics and engagement metrics. Paired *t*-tests and regression analyses will evaluate changes in the tobacco-related behaviors and attitudes of the participating students. Multiple linear regression analysis will be used to assess the strength and direction of the relationships between variables across the implementation period. As this study is exploratory, *p*-values will be interpreted alongside effect sizes and 95% confidence intervals. These will be the primary indicators of the strength and precision of the potential associations found. Specifically, Cohen’s d will be reported for paired *t*-tests, and standardized beta coefficients (β) for regression analyses. Statistical significance will be set to *p* < 0.05.

Interviews/focus groups will be audio-recorded, transcribed, and analyzed thematically by the MTs. More specifically, qualitative data will be analyzed using Braun and Clarke’s six-step framework for thematic analysis (Braun and Clarke) [50]. This involves transcribing and thematically analyzing the interviews and focus groups. First, the process involves verbatim transcription of audio-recorded interviews and focus groups, followed by multiple readings to understand the content. The transcripts will then be coded inductively (allowing themes to emerge) and deductively (focusing on study-specific aspects like adherence and quality of life). Codes will be grouped into categories, then refined into broader themes and subthemes. Finally, these themes will be presented with supporting quotes, and the qualitative findings will be triangulated with quantitative data to identify convergence or divergence between measurable outcomes and self-reported experiences [51].

## 3. Discussion

This protocol describes how the “Implementation Intentions” intervention will be adapted from the INTENT program [47] and implemented in Greece for the very first time among adolescents aged 13–16 years old within the FRESHAIR4Life study [37]. The aim is to reduce smoking initiation by engaging them with anti-smoking messages, and subsequently developing Personal Plans to predetermine specific responses to situations where they may be tempted to smoke. Towards that end, we will identify and educate three country MTs using IPCRG’s Teach-the-teacher model [43], enabling them to mentor volunteer teachers to conduct the two adapted INTENT school sessions. This implementation project is expected to achieve two main objectives: improving adolescents’ attitudes and actions regarding tobacco, and establishing a long-term, teacher-led program that can operate without external support.

Given that the “Implementation Intentions” intervention has not been previously adapted or implemented within Greece, its implementation in the Greek context will offer an innovative approach to preventing smoking among adolescents. Importantly, this approach has been proven as very effective in helping people do what they intend to do and in teaching them how to resist peer pressure and the urge to smoke, in other contexts as well [27,28,29,52]. In the UK, the original INTENT program, which our intervention is based on, utilized “If–Then” strategies; these strategies successfully reduced smoking among adolescents, leading to long-term effects on their susceptibility to smoking [42,47]. In addition, previous studies have shown the utility of “Implementation Intentions” in the reduction in TU and the prevention of smoking initiation, yielding favorable long-term results [53,54,55]. Consequently, by adapting “Implementation Intentions” to address local trends in TU and socioeconomic factors, our program will build upon the promising INTENT program and provide a novel, teacher-friendly intervention to assist Greek adolescents in resisting tobacco. Our planning approach (“Implementation Intentions”) has potential and offers a new direction compared to prior Greek school programs such as SmokeFreeGreece, which utilizes social learning and planned behavior theories, and “I do not smoke, I exercise,” which emphasizes exercise as a substitute for smoking. These programs primarily rely on educational sessions and awareness campaigns and lack a more personalized or individualized method to engage adolescents [40,56].

Our program will utilize the “Teach-the-Teacher” model, originally developed by the IPCRG, and adapted to train the country MTs to approach teachers and students, and adapt and “Implementation Intentions” to the Greek context [42,43]. This approach could support sustainability and scalability, empowering teachers to educate other teachers in the future so that more school teachers can start implementing the INTENT program, eliminating the need for constant external support. Studies have suggested that teacher-led, school-based prevention programs seem effective in preventing adolescents from smoking initiation and strengthening their refusal skills, especially when they are interactive, age-appropriate, and integrated into regular school activities [3,42,55,57]. Building on this, our intervention will be structured in two concise sessions, designed to fit the Greek academic calendar and avoid disruptions from holidays and exams due to appropriate planning. Moreover, we will utilize open digital platforms like Google Classroom to provide continuous support to teachers. On the other hand, our intervention could also empower teachers by improving their knowledge, attitudes and confidence towards preventing and reducing smoking among adolescents. These factors have been consistently highlighted by systematic reviews and meta-analyses to improve the delivery of health interventions in the classroom through structured training [47,55,57,58]. This multifaceted design may also provide broader psychosocial benefits, such as increased refusal confidence and reduced social isolation through group discussions, consistent with meta-analyses showing that “Implementation Intentions” strengthen behavioral intentions in adolescents [47,55,59]. Additionally, we anticipate that these improvements in participants’ knowledge, attitudes, and likelihood of smoking could be measured through the RE-AIM framework to determine reach, effectiveness, adoption, implementation, and maintenance.

This project could also help reduce adolescent tobacco use by training teachers to support their students in quitting smoking. Even though referrals require parents to get involved, this aspect broadens the program’s scope, moving beyond prevention to early intervention and assisting adolescents who might have initiated TU. Additionally, the intervention’s emphasis on teachers as guides could include training other community members, such as parents or youth advocates, to strengthen prevention initiatives [60,61,62]. These findings could add to the body of knowledge supporting combined tobacco control approaches, transitioning from standard awareness programs to proactive behavioral methods that consider social and environmental factors. This could then influence the wider use of tailored “Implementation Intentions” interventions within educational environments, not only in Europe, but also globally. Such programs, when adapted to specific cultures and implemented effectively, need few resources but significantly improve public health by possibly reducing the impact of tobacco-related diseases on local communities and providing adolescents with knowledge and skills that they could use throughout their lives.

### 3.1. Limitations

Our protocol outlines the adaptation and implementation plan of the INTENT program for smoking prevention for Greek adolescents. This intervention, to the best of our knowledge, will be the first to utilize “Implementation Intentions” in Greece to prevent smoking in adolescents. Nevertheless, several limitations should be considered. Relying on volunteer teachers could introduce selection bias and inconsistencies in program delivery, influenced by factors like teacher smoking status, workload, motivation or enthusiasm. In addition, the selected schools all have diverse socioeconomic student bodies, which could also lead to selection bias; thus, the findings might not be entirely applicable to rural or private schools. The condensed two-session format within a single school year, while practical, might not fully replicate the impact of the original eight-session program over four consecutive school years, potentially hindering long-term behavioral changes, given the limited window of observation. Although pilot testing will refine materials, unexpected cultural or language barriers could affect student engagement, particularly in diverse schools/settings. Existing anti-smoking initiatives within schools could also ‘dilute’ measurable results, though our program’s unique “If–Then” approach and tailored content could ‘stand out’ and integrate into current curricula. The three-month implementation period will assess initial outcomes but may not measure sustained effects. Another limitation of our study is the lack of a control group.

### 3.2. Dissemination Plan

The findings of this study will be disseminated to the scientific community through publications in international peer-reviewed journals. Additionally, the findings will be presented at national and/or international conferences, thereby making them accessible on relevant websites.

## Figures and Tables

**Table 1:** Outline of the schedule for enrollment, intervention, and assessment of the participating teachers and adolescents for this study based on the SPIRIT 2025 statement [41].

	STUDY PERIOD						
	Enrollment Teachers	Intervention Period Teachers		Enrollment Students	Intervention Period Students		Close-Out
TIMEPOINT *	0	t_1_	t_2_	t_3_	t_4_	t_5_	t_6_
ENROLLMENT:							
Eligibility screen	X						
Informed consent	X			X			
Assent form				X			
**INTERVENTIONS:**							
Online training session for teachers		X					
Live training sessions for teachers at six schools			X				
Delivery of Session 1 by teachers					X		
Delivery of Session 2 by teachers						X	
**ASSESSMENTS:**							
Pre-intervention questionnaires teachers and adolescents				X			
Post-intervention questionnaires teachers and adolescents							X
Semi-structured interviews and focus groups with teachers and adolescents							X

* t_1_, t_2_, t_3_, t_4_, t_5_, and t_6_ refer to the first, second, third, fourth, fifth, and sixth months of the study.

**Table 2:** Content of each part of the intervention for the teachers (training).

Part of the Intervention	Topic Covered	Goal	Key Components
**Session 1: Introductory Session**	Introduction to the adapted “Implementation Intentions” materials.	To provide the foundation for the training program, focusing on the theoretical underpinnings of the intervention, essential facilitation skills, and hands-on practice.	Share adapted materials, explain the goals of the intervention, the importance of tobacco prevention in youth, “If–Then” strategies, and answer questions.
**Session 2: Facilitation Skills Workshop**	Providing information on advanced facilitation skills for implementing the intervention.	To equip teachers with practical techniques and confidence to deliver the culturally adapted and evidence-based intervention.	Practice active listening, role-playing, deliver the “Implementation Intentions” curriculum, and receive feedback.

**Table 3:** Content of each part of the intervention for the students.

Part of the Intervention	Topic Covered	Goal	Key Components
**Session 1:** **Myths and Truths about smoking**	Introduction to Personal Plan (PP), factors influencing TU, myths about tobacco, long-term effects, resilience building	To introduce the program, educate on TU, debunk myths, and create a PP for refusing cigarettes.	Explain the goals of the intervention, the importance of TU prevention in adolescents, and “If–Then” strategies, and answer questions. Practice active listening, conduct role-playing, deliver the session, and receive feedback. Lead interactive discussion on myths and truths about tobacco use; quiz on long-term effects of smoking; development of each student’s personalized refusal strategy using “If–Then” statements; creation of each student’s Personal Plan.
**Session 2:** **Working on personal resilience**	Review of PP, motivation not to use tobacco products, personal goals, external influences, TU and stress, resilience strengthening	To reinforce the program, explore motivations and influences, and create another PP.	Review and reinforce the importance of a PP, explore motivation and non-TU motives, discuss the influence of personal goals on TU behaviors, and strengthen personal resilience.

**Table 4:** Study tools for students.

Type of Data	Measurement
**Quantitative data**	Baseline questionnaire for students
	Follow-up questionnaire for students
**Qualitative data**	Questions about participation and engagement in the sessions
	Questions about perceived impact

**Table 5:** Study tools for participating teachers.

Type of Data	Measurement
**Quantitative data**	Teacher self-assessment questionnaire
**Qualitative data**	Questions about participation and engagement in the sessions
	Questions about implementation phase assessment
	Questions about maintenance and scale-up

## Data Availability

No new data were created or analyzed in this study. Data sharing is not applicable to this article.

## Supplementary Materials

The following supporting information can be downloaded at https://www.mdpi.com/article/10.3390/healthcare14070938/s1, Table S1: SPIRIT 2025 checklist of items to address in a randomized trial protocol.
